# IL-37 Plays a Beneficial Role in Patients with Acute Coronary Syndrome

**DOI:** 10.1155/2019/9515346

**Published:** 2019-10-09

**Authors:** Xiaobo Mao, Ruirui Zhu, Fangyuan Zhang, Yucheng Zhong, Kunwu Yu, Yuzhen Wei, Haitao Sun, Wenbin Xu, Quan Luo, Yue Wang, Yan Ding, Qiutang Zeng

**Affiliations:** ^1^Department of Cardiology, Union Hospital, Tongji Medical College, Huazhong University of Science and Technology, Wuhan 430022, China; ^2^Department of Dermatology, Union Hospital, Tongji Medical College, Huazhong University of Science and Technology, Wuhan 430022, China

## Abstract

**Background:**

Interleukin-37 (IL-37) acts as an inhibitor of innate and adaptive immunity. However, the exact role of IL-37 in the patients with acute coronary syndrome (ACS) remains to be elucidated.

**Methods:**

Patients were classified into 4 groups: normal coronary artery (NCA), stable angina (SA), unstable angina (UA), and acute myocardial infarction (AMI). The circulating Treg, Th1, and Th17 frequencies were measured. The effect of IL-37 on stimulated peripheral blood mononuclear cells (PBMCs) and the influence of IL-37 on DCs were explored. In addition, the role of IL-37-treated tDCs on Treg cell expansion and the stability of these tDCs were also tested.

**Results:**

Our results showed that the circulating Treg frequencies were decreased, while Th1 and Th17 frequencies were increased in ACS patients, and that IL-37 expanded Tregs but suppressed Th1 and Th17 cells in activated PBMCs derived from ACS patients. Of note, IL-37-treated human DCs obtained a tolerogenic phenotype, and such tDCs promoted expansion of Tregs and decreased the Th1 and Th17 populations when cocultured with CD4^+^ T cells. Interestingly, IL-37-treated DCs from patients with ACS are phenotypically and functionally comparable to IL-37-treated DCs from NCA patients, and tolerogenic properties of IL-37-treated DCs were highly stable.

**Conclusion:**

In conclusion, our results reveal a beneficial role of IL-37 in the patients with ACS and suggest that autologous IL-37-treated tDCs may be a novel therapeutic strategy for the patients with ACS.

## 1. Introduction

It is well accepted that atherosclerosis (AS) is a chronic inflammatory disease and remains the principal cause of morbidity and mortality worldwide [1–3]. Acute coronary syndrome (ACS), including unstable angina (UA) and acute myocardial infarction (AMI), mainly resulted from plaque instability and is one of the leading causes for hospitalization [3]. Hence, it is important to explore the potential mechanisms involved in ACS and ultimately search for alternative therapeutic targets against it.

According to the cytokines they produce, CD4^+^ T cells are divided into different subsets, such as Th1, Th17, and regulatory T cells (Tregs). Previous studies have demonstrated that Th1 response contributes to plaque rupture and the onset of ACS, whereas in vivo inhibition of Th1 response reduces atherogenesis in an animal model [4, 5]. Interestingly, both clinical and experimental studies from our laboratory have found that Th17/Treg imbalance exists in patients with ACS and in ApoE^−/−^ mice [6, 7]. Therefore, these findings have implied that different CD4^+^ T cell subpopulations play an important role in plaque destabilization and the onset of ACS.

IL-37, termed as IL-1F7 previously, is a natural inhibitor of innate and adaptive immunity [8–11]. IL-37tg mice are protected from dextran sulfate sodium-induced colitis and LPS-induced septic shock, whereas treatment with human IL-37 plasmid-DNA ameliorates local and systemic inflammation in psoriasis and concanavalin A-induced hepatitis [8, 12–14]. Of note, our previous experimental researches revealed that IL-37 plays a beneficial part in myocardial infarction and myocardial ischemia/reperfusion injury [15, 16]. In spite of the previous study showing that plasma IL-37 is elevated in patients with ACS [17], the role of IL-37 in patients with ACS remains to be determined.

Dendritic cells (DCs) are professional antigen-presenting cells that regulate both immune response and peripheral tolerance [18–20]. It has been demonstrated that IL-10-treated DCs inhibit secretion of inflammatory cytokines and induce generation of regulatory T cells (Tregs) [21, 22]. Accumulating evidence indicates that specific antigen-loaded tDCs attenuate experimental autoimmune myocarditis and atherosclerosis [23, 24]. More importantly, our previous results show that IL-37 plus TNI-conditioned DCs obtained the characteristics of tolerogenic DCs (tDCs) and expanded the number of regulatory T cells, and adoptive transfer of these tDCs markedly ameliorated the infiltration of inflammatory cells in the infarct heart of mice [15]. Strikingly, a phase I study of autologous tolerogenic DCs in type 1 diabetic patients has been conducted, indicating that treatment with autologous dendritic cells is safe and well tolerated [25]. However, the utility of immunoregulatory tDCs as treatment for ACS patients is in the embryonic stage.

Many papers have reported that IL-37 transgenic mice exhibit less severe inflammation in models of myocardial infarction, endotoxin shock, colitis, lung, and spinal cord injury, while dendritic cells (DCs) from IL-37tg mice exhibit characteristics of tolerogenic DCs, and these mice have reduced antigen-specific responses [8, 12, 26]. Although these studies suggest the role of IL-37 in regulating immune responses, underlying mechanisms remain to be elucidated.

## 2. Materials and Methods

### 2.1. Reagents and Antibodies

Cell culture medium for PBMCs and DCs was RPMI 1640 (Gibco, Carlsbad, CA, USA) supplemented with 10% FCS (Gibco, Carlsbad, CA, USA) and 100 U/mL streptomycin/penicillin. Anti-human CD3, anti-human CD28, anti-human CD4-FITC, anti-human CD25-APC, anti-human Foxp3-PE, anti-human IFN-*γ*-PE, anti-human IL-17-PE, anti-human CD11c-APC/Cy7, anti-human HLA-DR-PerCp/Cy5.5, anti-human CD40-PE, and anti-human CD86-PE were all from eBioscience, San Diego, CA, USA. TRIzol, PrimeScript RT reagent kit, and SYBR Green Master Mix were from Takara Biotechnology, Dalian, China. Recombinant human GM-CSF and recombinant human IL-4 were obtained from PeproTech, Rocky Hill, NJ, USA. Meanwhile, human magnetic CD14 isolation kit and CD4 isolation kit were bought from Miltenyi Biotec, Auburn, CA, USA. Lipopolysaccharides (LPS) were from Sigma-Aldrich, St Louis, MO, USA, and recombinant human IL-37 was bought from AdipoGen AG, Liestal, Switzerland. Oxidised low density lipoprotein (oxLDL) was acquired from Yiyuan, Wuhan, China, and phorbol myristate acetate, ionomycin, and monensin were from Alexis Biochemicals, San Diego, CA, USA.

### 2.2. Patients

129 patients were recruited between December 2015 and July 2019 in the Department of Cardiology of Huazhong University of Science and Technology Affiliated Union Hospital. All the patients underwent diagnostic coronary angiography and were classified into 4 groups: (1) normal coronary artery (NCA), which included 30 subjects with normal coronary artery (21 men and 9 women, mean age 57.1 ± 10.1); (2) stable angina (SA) (18 men and 8 women, mean age 59.2 ± 10.3)—inclusion criteria: typical chest discomfort that was associated with horizontal or down-sloping ST-segment depression > 1 mm in an exercise test; (3) unstable angina (UA) (24 men and 11 women, mean age 58.3 ± 9.7)—inclusion criteria: chest pain at rest with definite ischemic electrocardiographic changes: ST-segment changes and/or T-wave inversions; and (4) acute myocardial infarction (AMI) (26 men and 12 women, mean age 58.2 ± 9.9)—inclusion criteria: myocardial infarction that was confirmed by a significant increase of troponin levels.

Patients with the following characteristics were excluded: valvular heart disease, thromboembolism, collagen disease, disseminated intravascular coagulation, severe liver and kidney disease, symptomatic heart failure, or malignant disease or who were on steroid therapy. All the patients were provided written informed consent prior to study entry, and the study was approved by the Ethics Committee of Tongji Medical College of Huazhong University of Science and Technology.

There was no significant difference in age, gender, smoking, history of hypertension, and diabetes in the four groups. The levels of C-reactive protein (CRP) in the ACS group (UA+AMI) were significantly higher than those in the non-ACS group (NCA+SA), and the cTNI and creatinine in the AMI group were higher than those in the other three groups. The other parameters including lipid and lipoprotein fractions, fasting glucose, and medications are listed in Supplementary [Supplementary-material supplementary-material-1].

### 2.3. Blood Samples

For the AMI patients, blood samples were acquired from the patients upon arrival into the department. For the rest of the study patients, fasting blood samples were obtained the next morning after admission. The samples were collected into sodium heparin Vacutainers (Becton-Dickinson). Peripheral blood mononuclear cells (PBMCs) were prepared by Ficoll density gradient for further use.

### 2.4. Generation of Human DCs

Human peripheral blood was obtained from NCA and ACS patients. CD14^+^ cells from PBMCs were isolated using a CD14 isolation kit. The purity of the isolated CD14^+^ monocytes was >0.95%. Immature DCs (imDCs) were generated from the CD14^+^ monocytes by culturing them in RPMI 1640 medium supplemented with 10% FCS, 40 ng/mL rhGM-CSF, 250 U/mL rhIL-4, and 100 U/mL penicillin/streptomycin for 5 days as previously described [27]. To induce mature DCs (mDCs), imDCs were incubated with 10 *μ*g/mL oxLDL for a further 48 hours. IL-37-treated human DCs (tDCs) were prepared by culturing imDCs with 10 *μ*g/mL oxLDL and 30 ng/mL IL-37 for 48 hours.

### 2.5. TCR Stimulation of PBMCs

PBMCs were resuspended at a density of 2 × 10^6^ cells/mL in RPMI 1640 medium supplemented with 100 U/mL penicillin/streptomycin, and 10% fetal calf serum (FCS). For TCR stimulations, PBMCs were activated with anti-CD3 (2 *μ*g/mL) and anti-CD28 (2 *μ*g/mL) in the presence or absence of 30 ng/mL IL-37 for 24 hours as previously described [28]. After 24 hours of culture, the cells were obtained to examine the percentages of Tregs, Th1 cells, and Th17 cells by flow cytometry. And Th1-, Th17-, and Treg-related gene expression levels were analyzed by RT-PCR.

### 2.6. Flow Cytometry

Flow cytometry was conducted as previously described [28]. In brief, for detection of CD4^+^CD25^+^Foxp3^+^ Treg cells, the cells were stained with anti-CD4-FITC and anti-CD25-APC and then stained with anti-Foxp3-PE after fixation and permeabilization. For analysis of Th1 and Th17 cells, PBMCs were stimulated with phorbol myristate acetate (20 ng/mL), ionomycin (1 *μ*g/mL), and monensin (2 *μ*mol/mL) for 4 hours. Fixation and permeabilization were necessary before staining with IFN-*γ* or IL-17 antibody. For analysis of the characterization for cultured DCs, the cells were stained with anti-CD11c-APC/Cy7, anti-HLA-DR-PerCp/Cy5.5, anti-CD40-PE, or anti-CD86-PE for 30 minutes.

PBMCs were obtained from NCA patients and resuspended at a density of 2 × 10^6^ cells/mL in RPMI 1640 medium. These cells were cultured for 5 days at 37°C with 5.0% CO_2_ supplemented with 2 mL serum of NCA, SA, UA, and AMI patients. Finally, PBMCs were activated with anti-CD3 (2 *μ*g/mL) and anti-CD28 (2 *μ*g/mL) for 24 hours. The cells were obtained to examine the percentages of Tregs, Th1 cells, and Th17 cells by flow cytometry.

### 2.7. RT-PCR Analysis

Total RNA was extracted from cells using TRIzol (Takara Biotechnology, Dalian, China) and reverse transcribed into cDNA using the PrimeScript RT reagent kit (Takara Biotechnology, Dalian, China), and the mRNA levels of the target genes were quantified using SYBR Green Master Mix (Takara Biotechnology) with an ABI PRISM 7900 Sequence Detector System (Applied Biosystems, Foster City, CA, USA) as previously described [23]. Each reaction was performed in triplicate, and changes in the relative gene expression level normalized to the GAPDH level were calculated using the relative threshold cycle method. The primer sequences are shown in [Supplementary-material supplementary-material-1].

### 2.8. DC/T Cell Coculture

After washing with PBS 3 times, imDCs, tDCs, or mDCs (2 × 10^5^ cells/mL) were cocultured with CD4^+^ T cells (1 × 10^6^ cells/mL) isolated from PBMCs. The mixed cells were cultured for 5 days at 37°C with 5.0% CO_2_ in 2 mL RPMI 1640 supplemented with 10% FCS.

### 2.9. Stability of IL-37-Treated DCs

Phenotypic stability of IL-37-treated DCs (tDCs) was determined in response to inflammatory stimulation. imDCs, tDCs, and mDCs were washed, adjusted to 5 × 10^5^ cells/mL per well, and cultured in RPMI 1640 medium supplemented with 10% FCS in the presence or absence of LPS (0.1 *μ*g/mL) for 1 day. After 24 hours, DCs were analyzed for cell surface phenotype by flow cytometry and the mRNA levels of IL-12, IL-10, and TGF-*β* in different DC groups were determined by RT-PCR.

### 2.10. Statistical Analysis

All of the data were given as the mean ± SD. Comparisons between 2 groups were performed by Student's *t*-test. For comparisons ≥3 groups, one-way ANOVA followed by Newman-Keuls post hoc test was used. All analyses were done using GraphPad Prism 6.0 (GraphPad Software, Inc., La Jolla, CA), and *P* < 0.05 was considered to be statistically significant.

## 3. Results

### 3.1. The Circulating Treg Frequencies Were Decreased, While Th1 and Th17 Frequencies Were Increased in ACS Patients

**Figure 1:**
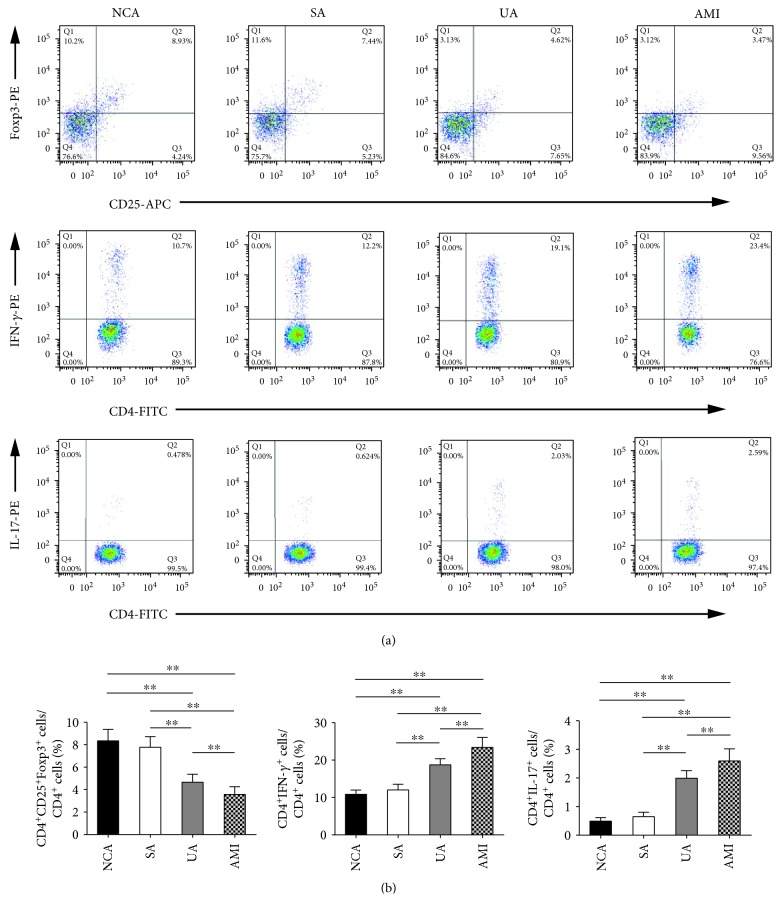
The circulating Treg frequencies were decreased, while Th1 and Th17 frequencies were increased in ACS patients. (a) Representative images of Tregs, Th1 cells, and Th17 cells in the four groups are shown. (b) Results of statistical analysis of average percentages of Tregs, Th1 cells, and Th17 cells. All data are expressed as mean ± SEM, and one-way ANOVA was followed by a post hoc Student-Newman-Keuls test. NCA (*n* = 30), SA (*n* = 26), UA (*n* = 35), and AMI (*n* = 38). ∗∗ indicates *P* < 0.01. NCA: normal coronary artery; SA: stable angina; UA: unstable angina; AMI: acute myocardial infarction.

As shown in Figure 1, the frequencies of Tregs (CD4^+^CD25^+^Foxp3^+^/CD4^+^ T cells) were markedly lower in the UA group (4.65 ± 0.72%) and AMI group (3.57 ± 0.68%) compared with the NCA group (8.35 ± 1.01%) (*P* < 0.01), while Treg frequencies in the UA and AMI groups were considerably lower than that in the SA group (7.77 ± 0.95%) (*P* < 0.01). This is consistent with previous research [6]. However, Treg frequencies in the NCA and SA groups were similar (*P* > 0.05). The frequencies of Th1 (CD4^+^IFN-*γ*^+^/CD4^+^ T cells) were markedly higher in patients with UA (18.73 ± 1.63%) and AMI (23.38 ± 2.68%) than those with NCA (10.87 ± 1.11%) and SA (12.04 ± 1.49%) (*P* < 0.01), while those data in the NCA and SA groups were similar (*P* > 0.05) (Figures 1(a) and 1(b)). In addition, the percentages of Th17 (CD4^+^IL-17^+^/CD4^+^ T cells) were substantially increased in patients with UA (1.99 ± 0.27%) and AMI (2.60 ± 0.42%) than those with NCA (0.49 ± 0.12%) and SA (0.65 ± 0.16%) (*P* < 0.01). However, there was no obvious difference between the NCA and the SA group (*P* > 0.05) (Figures 1(a) and 1(b)). The number of CD4^+^ cells in the four groups was shown in [Supplementary-material supplementary-material-1]. Together, these results indicate that the circulating Treg frequencies were decreased in ACS patients, while Th1 and Th17 frequencies were increased.

We next quantified Th1-, Th17-, and Treg-related gene expression levels in these four groups. As shown in [Supplementary-material supplementary-material-1], real-time PCR analysis suggested that the expressions of T-bet, IFN-*γ*, ROR*γ*t, and IL-17 were considerably higher in patients with UA and AMI than those with NCA and SA (all *P* < 0.01), while mRNA levels of Foxp3, IL-10, and TGF-*β* were significantly lower in patients with UA and AMI than those with NCA and SA (all *P* < 0.01). Furthermore, all the gene expressions mentioned above were similar between the NCA and SA groups ([Supplementary-material supplementary-material-1]).

Recently, several clinical experiments have showed that hs-CRP is not only a biomarker of inflammations but also a marker of atheromatous plaque vulnerability [29–31]. In line with these findings, our study also revealed that the levels of CRP in the ACS patients were significantly higher than those in the NCA and SA patients ([Supplementary-material supplementary-material-1]). Meanwhile, we found that TNI levels were positively correlated with CRP for the AMI patients ([Supplementary-material supplementary-material-1]).

### 3.2. The Effect of IL-37 on Stimulated Peripheral Blood Mononuclear Cells (PBMCs)

To detect the role of IL-37 on activated PBMCs, anti-CD3 and anti-CD28 antibodies were used. Among the concentrations of 10, 30, and 100 ng/mL, 30 ng/mL IL-37 was the optimal one, resulting in strong upregulation of Treg frequencies and inhibition of Th1 and Th17 frequencies. Therefore, we used 30 ng/mL IL-37 in this study.

**Figure 2:**
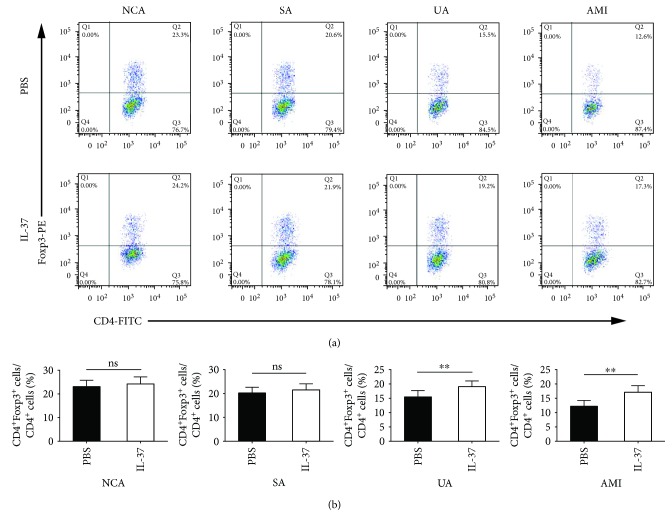
IL-37 significantly upregulated the frequencies of Tregs in the UA group and the AMI group compared with PBS-treated activated PBMCs. PBMCs were activated by anti-CD3 and anti-CD28 antibodies for 24 h in the presence or absence of 30 ng/mL IL-37. (a) Representative images of Tregs (CD4^+^Foxp3^+^/CD4^+^ T cells) are shown. (b) Results of statistical analysis of average percentages of Tregs. All data are expressed as mean ± SEM, and differences were evaluated using Student *t*-test. NCA (*n* = 30), SA (*n* = 26), UA (*n* = 35), and AMI (*n* = 38). ∗∗ indicates *P* < 0.01. ns: not significant; NCA: normal coronary artery; SA: stable angina; UA: unstable angina; AMI: acute myocardial infarction.

**Figure 3:**
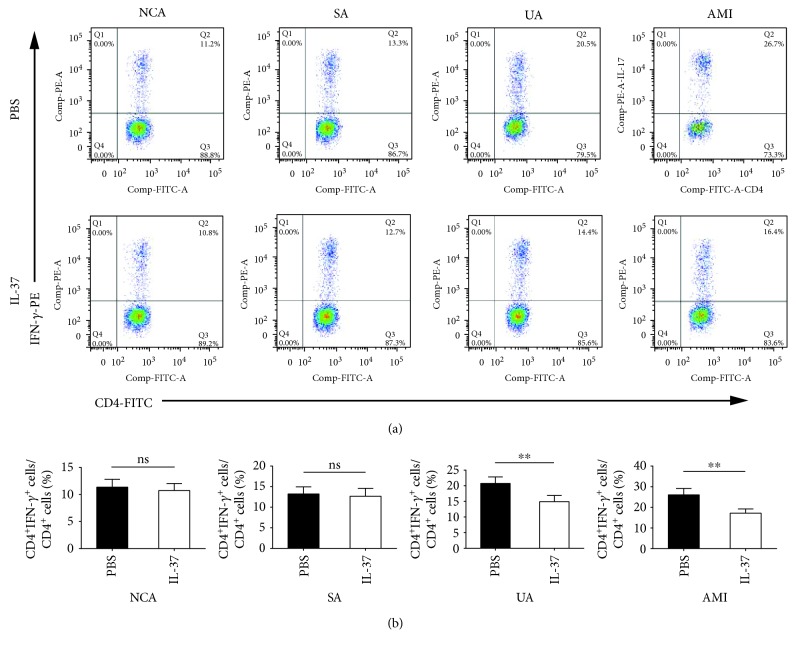
IL-37 significantly decreased the percentages of Th1 cells in the UA group and the AMI group compared with PBS-treated activated PBMCs. PBMCs were activated by anti-CD3 and anti-CD28 antibodies for 24 h in the presence or absence of 30 ng/mL IL-37. (a) Representative images of Th1 cells are shown. (b) Results of statistical analysis of average percentages of Th1 cells. All data are expressed as mean ± SEM, and differences were evaluated using Student *t*-test. NCA (*n* = 30), SA (*n* = 26), UA (*n* = 35), and AMI (*n* = 38). ∗∗ indicates *P* < 0.01. ns: not significant; NCA: normal coronary artery; SA: stable angina; UA: unstable angina; AMI: acute myocardial infarction.

**Figure 4:**
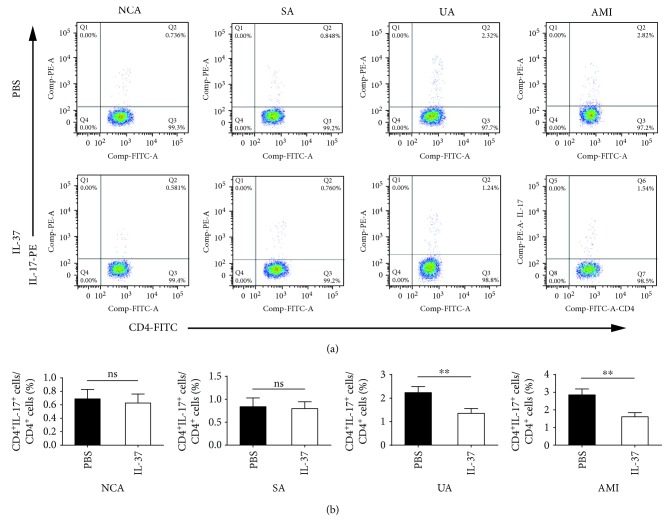
IL-37 significantly decreased the percentages of Th17 cells in the UA group and the AMI group compared with PBS-treated activated PBMCs. PBMCs were activated by anti-CD3 and anti-CD28 antibodies for 24 h in the presence or absence of 30 ng/mL IL-37. (a) Representative images of Th17 cells are shown. (b) Results of statistical analysis of average percentages of Th17 cells. All data are expressed as mean ± SEM, and differences were evaluated using Student *t*-test. NCA (*n* = 30), SA (*n* = 26), UA (*n* = 35), and AMI (*n* = 38). ∗∗ indicates *P* < 0.01. ns: not significant; NCA: normal coronary artery; SA: stable angina; UA: unstable angina; AMI: acute myocardial infarction.

Compared with PBS-treated PBMCs, IL-37 significantly upregulated the frequencies of Tregs (CD4^+^Foxp3^+^/CD4^+^ T cells) in the UA group (19.04 ± 1.99% vs. 15.52 ± 2.21%, *P* < 0.01) and the AMI group (17.10 ± 2.29% vs. 12.23 ± 2.00%, *P* < 0.01), while IL-37 had no effect on the percentages of Tregs in the NCA group (23.13 ± 2.62% vs. 24.16 ± 3.04%, *P* > 0.05) and the SA group (20.27 ± 2.33% vs. 21.48 ± 2.58%, *P* > 0.05) (Figure 2). As shown in Figure 3, the frequencies of Th1 (CD4^+^IFN-*γ*^+^/CD4^+^ T cells) were markedly reduced by IL-37 treatment in the UA group (14.93 ± 1.97% vs. 20.74 ± 2.08%, *P* < 0.01) and the AMI group (17.19 ± 2.08% vs. 26.09 ± 3.07%, *P* < 0.01), while the proportions of Th1 cells were not affected by IL-37 treatment in the NCA group and the SA group. Similar to the frequencies of Th1 cells, administration of IL-37 considerably decreased the proportions of Th17 (CD4^+^IL-17^+^/CD4^+^ T cells) in the UA group (1.35 ± 0.21% vs. 2.24 ± 0.26%, *P* < 0.01) and the AMI group (1.61 ± 0.23% vs. 2.85 ± 0.34%, P < 0.01), while the proportions of Th17 cells were not affected by IL-37 in the NCA group and the SA group (Figure 4).

Interestingly, we also found that PBMCs cultured with serum from UA or AMI patients significantly downregulated the frequencies of Tregs (CD4^+^Foxp3^+^/CD4^+^ T cells) and markedly increased the frequencies of Th1 (CD4^+^IFN-*γ*^+^/CD4^+^ T cells) and the proportions of Th17 (CD4^+^IL-17^+^/CD4^+^ T cells) compared with PBMCs cultured with serum from NCA or SA patients ([Supplementary-material supplementary-material-1]).

We next measured the effect of IL-37 on Th1-, Th17-, and Treg-related gene expression levels in these four groups. As shown in [Supplementary-material supplementary-material-1], real-time PCR analysis indicated that the expressions of T-bet, IFN-*γ*, ROR*γ*t, and IL-17 were considerably inhibited by IL-37 in the UA and AMI groups (all *P* < 0.01), while mRNA levels of Foxp3, IL-10, and TGF-*β* were significantly expanded by IL-37 in the UA and AMI groups (all *P* < 0.01). Furthermore, treatment with IL-37 showed no influence on all the gene expressions mentioned above in the NCA and SA groups ([Supplementary-material supplementary-material-1]). In sum, these results suggest that IL-37 expands Tregs and Treg-associated gene expression levels but suppresses Th1 cells, Th17 cells, and their related gene expression levels in activated PBMCs derived from ACS patients.

### 3.3. IL-37-Treated Human DCs Show a Tolerogenic Phenotype

We next investigate the influence of IL-37 on DCs. Oxidised low density lipoprotein (oxLDL) was chosen as a positive control, for oxLDL played a central role in the progression of atherosclerosis and was capable of promoting DC maturation [27, 32]. IL-37-treated human DCs were prepared by culturing imDCs with oxLDL for 48 h in the presence of IL-37.

**Figure 5:**
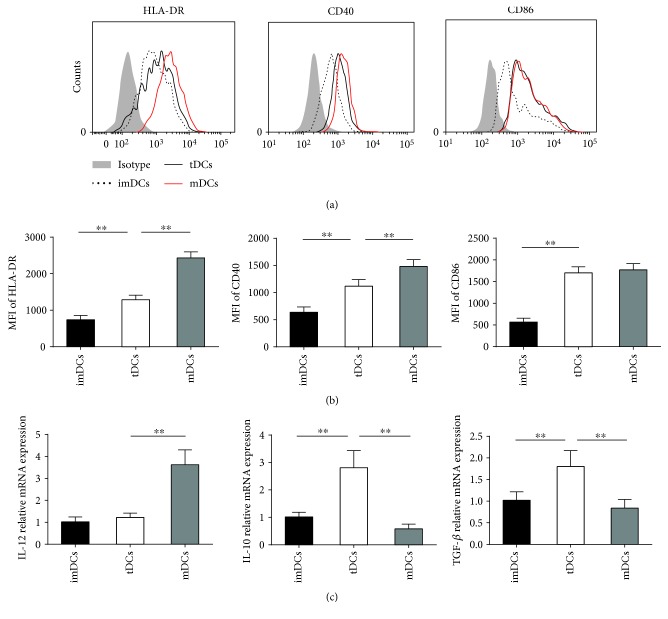
IL-37-treated human DCs display a tolerogenic phenotype. Monocyte-derived DCs from ACS patients were cultured in the absence of stimulus (immature DCs (imDCs)) or in the presence of 10 *μ*g/mL oxLDL (mature DCs (mDCs)) or 30 ng/mL IL-37 plus 10 *μ*g/mL oxLDL (tolerogenic DCs (tDCs)). (a) DCs were stained with isotype control antibodies or with specific antibodies against HLA-DR, CD40, and CD86 and analyzed by fluorescence-activated cell sorting. (b) Mean fluorescence intensities (MFIs) for HLA-DR, CD40, and CD86 were quantified. (c) Analysis of the mRNA levels of IL-12, IL-10, and TGF-*β* in different DCs groups. All data are expressed as mean ± SEM (*n* = 6/experimentand three experiments were performed), and one-way ANOVA was followed by a post hoc Student-Newman-Keuls test. ∗∗ indicates *P* < 0.01.

First, we compared the surface markers of imDCs, IL-37-treated DCs, and mDCs. As expected, untreated monocyte-derived DCs expressed low levels of HLA-DR, CD40, and CD86, a characteristic of imDCs (Figure 5(a)), and further maturation with oxLDL increased levels of these surface markers mentioned above (Figure 5(a)), a characteristic of mDCs. Compared with mDCs, IL-37-treated DCs showed lower mean fluorescence intensity (MFI) of HLA-DR molecules and CD40 (Figures 5(a) and 5(b)). However, the level of CD86 was comparable between IL-37-treated DCs and mDCs. As shown in Figure 5(c), results showed that IL-37-treated DCs produced lower mRNA levels of IL-12, as well as higher mRNA levels of IL-10 and TGF-*β*, than the mDC subset. Together, these results indicate that IL-37-treated DCs obtain a tolerogenic phenotype.

We next measured MFI of DC-related cell surface markers of DCs from NCA, UA, and AMI patients. To our surprise, IL-37-treated DCs from all these three groups showed similar MFI of HLA-DR, CD40, and CD86 and displayed comparable mRNA levels of IL-12, IL-10, and TGF-*β* ([Supplementary-material supplementary-material-1]). These results suggest that IL-37-treated DCs from patients with ACS are phenotypically and functionally comparable to IL-37-treated DCs from NCA patients.

We have previously demonstrated that IL-37 suppresses TLR-4 expression in murine myocardial ischemia/reperfusion injury [16]. As expected, we also found that the relative mRNA expressions of TLR-4 were lower in tDCs than in mDCs ([Supplementary-material supplementary-material-1]).

### 3.4. TDCs Have a Potential to Induce Tolerance

**Figure 6:**
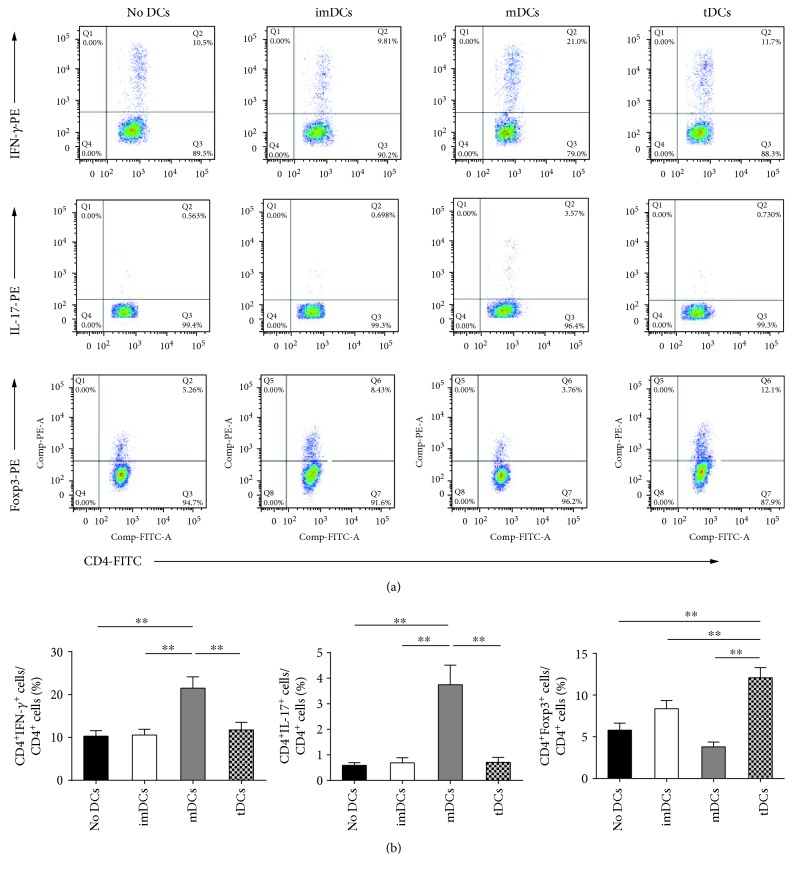
Tolerogenic dendritic cells (tDCs) have a potential to induce tolerance in vitro. Human CD4^+^ T cells (1 × 10^6^ cells/mL) from ACS patients were cultured for 5 days in the presence of medium alone (no DCs) or combined with immature DCs (imDCs), mature DCs (mDCs), or tDCs (2 × 10^5^ cells/mL). The cells were labeled 5 days later with anti-CD4, anti-CD25, anti-IFN-*γ*, anti-IL-17, and anti-Foxp3 and analyzed by fluorescence-activated cell sorting (FACS). (a) CD4^+^ T cell subsets were gated, and representative images of Th1, Th17, and Tregs are shown. (b) Graphs represent the average percentages of Th1, Th17, and Tregs in human CD4^+^ T cells of different groups. All data are expressed as mean ± SEM (*n* = 5/experiment and three experiments were performed), and one-way ANOVA was followed by a post hoc Student-Newman-Keuls test. ∗∗ indicates *P* < 0.01.

To detect the role of tDCs on CD4^+^T cells, a coculture research was executed. Compared to imDCs and mDCs, IL-37-treated tDCs significantly augmented the CD4^+^Foxp3^+^ Treg cell population (Figures 6(a) and 6(b)) and the tDC group showed enhanced mRNA expression of Foxp3, IL-10, and TGF-*β* ([Supplementary-material supplementary-material-1]). Contrary to the mDC subset, tDCs decreased the CD4^+^IFN-*γ*^+^ T cell and CD4^+^IL-17^+^ T cell populations and inhibited the mRNA levels of IFN-*γ* and IL-17 (Figures 6(a) and 6(b) and [Supplementary-material supplementary-material-1]). Taken together, these results suggest that IL-37-treated tDCs have ability to promote tolerance.

### 3.5. Tolerogenic Properties of IL-37-Treated Human DCs Are Highly Stable

**Figure 7:**
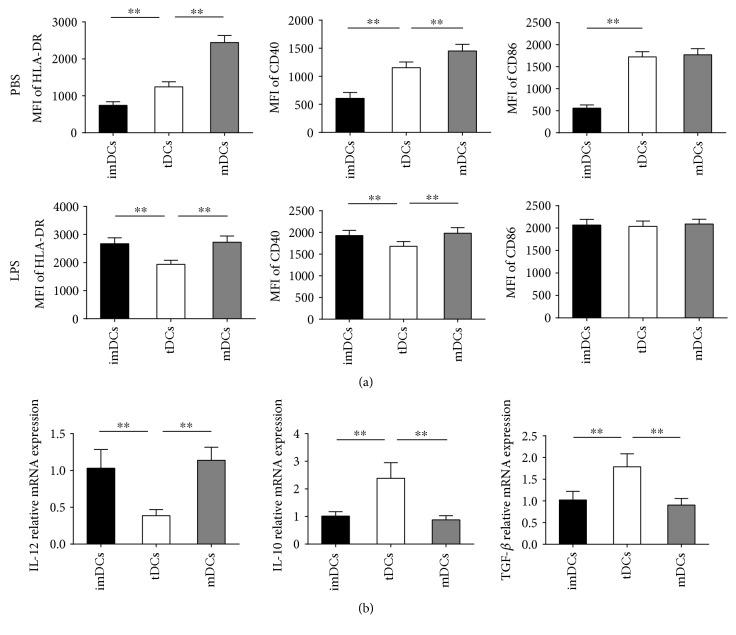
Tolerogenic properties of IL-37-treated human DCs are highly stable. After removal of IL-37 and oxLDL, imDCs, mDCs, or IL-37-treated DCs (tDCs) were cultured in the absence or presence of LPS (0.1 *μ*g/mL). After stimulation for 24 hours, DCs were analyzed for cell surface phenotype by flow cytometry. (a) Mean fluorescence intensities (MFIs) for HLA-DR, CD40, and CD86 were quantified. (b) Analysis of the mRNA levels of IL-12, IL-10, and TGF-*β* in different DC groups after LPS stimulation for 24 hours. All data are expressed as mean ± SEM (*n* = 3/experiment and four experiments were performed), and one-way ANOVA was followed by a post hoc Student-Newman-Keuls test. ∗∗ indicates *P* < 0.01.

In vivo, after encountering inflammation mediators, whether these in vitro-generated tDCs switch to mDCs is a potential risk. To address this issue, the stability of tDCs was tested by stimulation with LPS for 24 hours after removal of IL-37. The surface phenotype of DCs and mRNA levels of cytokine production were compared. As shown in Figure 7(a), the expressions of HLA-DR and CD40 on tDCs were increased only minimally and remained low, whereas the expression of CD86 was similar between iDCs, tDCs, and mDCs. In contrast, iDCs matured and displayed upregulated expression of costimulatory molecules of HLA-DR, CD40, and CD86 on addition of LPS for 24 hours. Furthermore, mRNA levels of both IL-10 and TGF-*β* in tDCs were significantly higher than those in iDCs and mDCs after stimulation of LPS, whereas mRNA productions of IL-12 in tDCs were markedly lower than those in iDCs and mDCs after LPS activation (Figure 7(b)). The above findings indicated that IL-37-treated DCs are refractory to stimulation with LPS. Hence, their tolerogenic properties may be highly stable even after encountering inflammation mediators.

## 4. Discussion

We showed in the present study that the circulating Treg frequencies were decreased, while Th1 and Th17 frequencies were increased in ACS patients and that IL-37 expanded Tregs but suppressed Th1 and Th17 cells in activated PBMCs derived from ACS patients. Of note, we showed for the first time that IL-37-treated human DCs obtained a tolerogenic phenotype and such tDCs promoted expansion of Tregs and decreased the Th1 and Th17 populations. Interestingly, IL-37-treated DCs from patients with ACS are phenotypically and functionally comparable to IL-37-treated DCs from NCA patients, and tolerogenic properties of IL-37-treated DCs were demonstrated to be highly stable. Taken together, these results indicate that IL-37 displays a therapeutic potential in the patients with ACS.

Inflammation plays a central part in ACS, which is a consequence of coronary plaque erosion or rupture [1–3]. Recently, several clinical experiments have showed that hs-CRP is not only a biomarker of inflammations but also a marker of atheromatous plaque vulnerability [29–31]. In line with these findings, our study also revealed that the levels of hs-CRP in the ACS patients were significantly higher than those in the NCA patients. Meanwhile, we also found that TNI levels were positively correlated with hs-CRP for the AMI patients. Previous publications have reported that inhibition of Th1 response in vivo reduces atherogenesis in an animal model, while Th1 response contributes to plaque rupture and the onset of ACS [4, 5]. Moreover, accumulating evidence has shown that CD4^+^CD25^+^ Treg deficiency enhanced atherosclerotic lesion development in LDLR^−/−^ mice, whereas adoptive transfer of CD4^+^CD25^+^ Tregs attenuated the initiation and progression of atherosclerosis in ApoE^−/−^ mice [33, 34]. Importantly, clinical and experimental studies from our laboratory have found that Th17/Treg imbalance exists in patients with ACS and in ApoE^−/−^ mice, revealing that Th17/Treg imbalance may take a part in plaque destabilization and the onset of ACS [6, 7]. Hence, these studies have demonstrated that Th1, Th17, and Tregs play an important role in plaque destabilization and the onset of ACS. Previous studies have showed that IL-37 is also involved in the improvement of inflammation due to mast cell activation induced by fungi and the inhibition of proinflammatory IL-1 family members and TNF by this inhibitory cytokine in fibromyalgia could have a therapeutic effect [35, 36]. Strikingly, our recent study revealed that IL-37 significantly increased the frequencies of Tregs and decreased the frequencies of Th1 and Th17 cells in ApoE^−/−^ mice [37]. In line with this publication, we found that circulating Treg frequencies were decreased, while Th1 and Th17 frequencies were increased in ACS patients, and that IL-37 expanded Tregs but suppressed Th1 and Th17 cells in activated PBMCs derived from ACS patients. Therefore, we speculate that IL-37 plays a regulatory role in the patients with ACS via its effects on Tregs, Th1 cells, and Th17 cells.

Apart from the CD4^+^ T cell subpopulations mentioned above, different cytokines also have complicated effects on atherosclerosis or ACS. IFN-*γ* is a proinflammatory cytokine highly expressed in atherosclerotic lesions, while ACS patients display significantly higher IL-17 and IL-17-induced cytokines (such as IL-6 and IL-8) [38, 39]. Indeed, previous studies clarified that TGF-*β* is a stabilizing factor in human atherosclerotic plaques and IL-10 knockout or IL-10 transgene could significantly exacerbate or ameliorate the development of atherosclerosis [40, 41]. In the present study, we also found that the expressions of IFN-*γ* and IL-17 were considerably higher in patients with UA and AMI than in those with NCA and SA, while mRNA levels of IL-10 and TGF-*β* were significantly lower in patients with UA and AMI than in those with NCA and SA. Interestingly, IL-37 suppressed IFN-*γ* and IL-17 mRNA expression levels but expanded IL-10 and TGF-*β* gene expression levels in activated PBMCs derived from ACS patients. Consequently, our results again confirmed that IL-37 may exert a therapeutic effect in the patients with ACS via decreasing the expression of proinflammatory cytokines (IFN-*γ* and IL-17) and increasing the levels of anti-inflammatory cytokines (IL-10 and TGF-*β*).

It has been demonstrated that fully mature DCs are immunogenic and semimature DCs are tolerogenic [20]. Different from immunogenic mDCs, the role of these tDCs are regulated by more secretion of immunosuppressive cytokines, decreased expression of IL-12, and reduced levels of costimulatory molecules and MHC-II, hence inducing expansion of Tregs and delivering inadequate signals for effector T cell activation [42–44]. Of note, several publications reported that DCs treated with IL-10 and ApoB100 ameliorate atherosclerosis, while IL-37tg mice-derived BMDCs and splenic DCs showed reduced expression of costimulatory molecules and MHC-II after treatment with LPS [8, 9, 24]. Recently, our group found that DCs treated with IL-37 plus troponin I alleviate cardiac remodeling after myocardial infarction [15]. In addition, Liu et al. reported that IL-37 attenuated the maturation of DCs through the IL-1R8-TLR4-NF-*κ*B pathway in ApoE^−/−^ mice [45]. These two recent publications indicated that IL-37 could ameliorate atherosclerosis and cardiac remodeling after myocardial infarction in the murine model via targeting DCs. Consistent with these findings, we showed that IL-37-treated human DCs displayed lower MFI of HLA-DR molecules and CD40 and produced lower mRNA levels of IL-12, as well as higher mRNA levels of IL-10 and TGF-*β* than mDCs. These results suggest that DCs treated with IL-37 and oxLDL for 48 hours obtain the character of tDCs. Moreover, CD4^+^ T cells were cocultured with IL-37-treated tDCs to investigate the role of these tDCs in vitro. Our data showed that tDCs markedly increased the CD4^+^Foxp3^+^ Treg cell population and Foxp3 mRNA expression, enhanced mRNA expression of IL-10 and TGF-*β*, decreased the Th1 and Th17 populations, and inhibited the mRNA levels of IFN-*γ* and IL-17 compared with mDCs. These results together reveal that IL-37-treated tDCs have the capacity to induce tolerance when cocultured with CD4^+^ T cells.

In spite of clinical trials using immunogenic DC for cancer therapy having evolved over 2 decades [46, 47], treatment with tDCs is just emerging in the clinical arena. Firstly and very importantly, a potential risk of the utility of donor tDCs is the destruction of the injected cells by the immune system of the recipient [48]. Hence, using autologous tDCs will minimize this risk. As DCs have been implicated in atherosclerosis pathogenesis [49], we investigate whether IL-37 could be used for the establishment of tDCs from patients with ACS. In the present study, we surprisingly found that IL-37-treated DCs from patients with ACS are phenotypically and functionally comparable to IL-37-treated DCs from NCA patients, indicating that it is still possible to generate tDCs ex vivo for autologous cellular immunotherapy. To our knowledge, this is the first study to investigate the possibility of generation of tDCs from ACS patients. Another essential issue is whether in vitro-generated tDCs are stable and refractory to maturation in vivo, as the maturation-sensitive tDCs in ongoing inflammatory disease have the potential to convert tDCs into mature DCs and eventually aggravate disease [50]. In this study, the stability of tDCs was tested by stimulation with LPS for 24 hours after removal of IL-37. We have demonstrated that IL-37-treated DCs are refractory to stimulation with LPS, a phenomenon previously termed as LPS desensitisation [51], indicating that the tolerogenic properties of IL-37-treated tDCs are highly stable. Taken together, these findings suggest that autologous IL-37-treated tDCs may be a novel therapeutic strategy for the patients with ACS in the future.

In summary, our data demonstrate that IL-37 expanded Tregs but suppressed Th1 and Th17 cells in activated PBMCs derived from ACS patients and that IL-37-treated human DCs acquired a tolerogenic phenotype and such tDCs promoted expansion of Tregs and decreased the Th1 and Th17 populations. In addition, IL-37-treated DCs from patients with ACS are phenotypically and functionally comparable to IL-37-treated DCs from NCA patients, and these tolerogenic properties of IL-37-treated DCs were highly stable. Therefore, the present study provides the evidence that IL-37 exerts the anti-inflammatory role in vitro and suggests that autologous IL-37-treated tDCs may be a novel therapeutic strategy for the patients with ACS. However, two limitations of the present study should be considered. Firstly, the number of study patients is relatively small and our conclusions need to be proven in a larger scale of the population. Secondly, two previous studies have demonstrated that the anti-inflammatory role of IL-37 requires the receptors IL-18Ra and TIR-8 (SIGIRR) [52, 53]. Hence, IL-18Ra and TIR-8/SIGIRR knockout mice are required to further investigate the molecular mechanisms of this interaction in plaque progression and destabilization.

## Data Availability

The data used to support the findings of this study are available from the corresponding author upon request.

## Supplementary Materials

Supplementary Materials Figure S1: the number of CD4^+^ cells in the four groups. All data are expressed as mean ± SEM, and one-way ANOVA was followed by a post hoc Student-Newman-Keuls test. NCA (*n* = 30), SA (*n* = 26), UA (*n* = 35), and AMI (*n* = 38). ∗ indicates *P* < 0.05 and ^∗∗^ indicates *P* < 0.01. NCA: normal coronary artery; SA: stable angina; UA: unstable angina; AMI: acute myocardial infarction. Figure S2: Th1-, Th17-, and Treg-related gene expression levels in the four groups. The mRNA expressions of T-bet, IFN-*γ*, ROR*γ*t, IL-17, Foxp3, IL-10, and TGF-*β* are shown. All data are expressed as mean ± SEM, and one-way ANOVA was followed by a post hoc Student-Newman-Keuls test. NCA (*n* = 30), SA (*n* = 26), UA (*n* = 35), and AMI (*n* = 38). ∗ indicates *P* < 0.05 and ^∗∗^ indicates *P* < 0.01. NCA: normal coronary artery; SA: stable angina; UA: unstable angina; AMI: acute myocardial infarction. Figure S3: TNI levels were positively correlated with CRP for the AMI patients (*n* = 38). AMI: acute myocardial infarction. Figure S4: PBMCs cultured with serum from UA or AMI patients significantly downregulated the frequencies of Tregs (CD4^+^Foxp3^+^/CD4^+^ T cells) and markedly increased the frequencies of Th1 (CD4^+^IFN-*γ*^+^/CD4^+^ T cells) and the proportions of Th17 (CD4^+^IL-17^+^/CD4^+^ T cells) compared with PBMCs cultured with serum from NCA or SA patients. All data are expressed as mean ± SEM, and one-way ANOVA was followed by a post hoc Student-Newman-Keuls test. NCA (*n* = 10), SA (*n* = 10), UA (*n* = 10), and AMI (*n* = 10). ∗∗ indicates *P* < 0.01. ns: not significant; NCA: normal coronary artery; SA: stable angina; UA: unstable angina; AMI: acute myocardial infarction. Figure S5: the effect of IL-37 on Th1-, Th17-, and Treg-related gene expression levels in activated PBMCs. The mRNA expressions of T-bet, IFN-*γ*, ROR*γ*t, IL-17, Foxp3, IL-10, and TGF-*β* are shown. All data are expressed as mean ± SEM, and differences were evaluated using Student's *t*-test. NCA (*n* = 30), SA (*n* = 26), UA (*n* = 35), and AMI (*n* = 38). ∗∗ indicates *P* < 0.01. ns: not significant; NCA: normal coronary artery; SA: stable angina; UA: unstable angina; AMI: acute myocardial infarction. Figure S6: IL-37-treated DCs from patients with ACS are phenotypically and functionally comparable to IL-37-treated DCs from NCA patients. (A) Mean fluorescence intensities (MFIs) for HLA-DR, CD40, and CD86 were quantified. (B) Analysis of the mRNA levels of IL-10, TGF-*β*, and IL-12 in different patient groups. All data are expressed as mean ± SEM, and one-way ANOVA was used. NCA (*n* = 6), UA (*n* = 8), and AMI (*n* = 8). ns: not significant; NCA: normal coronary artery; UA: unstable angina; AMI: acute myocardial infarction. Figure S7: TLR-4 relative mRNA expressions in the three groups were shown. All data are expressed as mean ± SEM (*n* = 6/experiment and three experiments were performed), and one-way ANOVA was followed by a post hoc Student-Newman-Keuls test. ∗∗ indicates *P* < 0.01. imDCs: immature DCs; mDCs: mature DCs; tDCs: tolerogenic DCs. Figure S8: analysis of the mRNA levels of Foxp3, IL-10, TGF-*β*, IFN-*γ*, and IL-17 in different groups. All data are expressed as mean ± SEM (*n* = 5/experiment and three experiments were performed), and one-way ANOVA was followed by a post hoc Student-Newman-Keuls test. ∗∗ indicates *P* < 0.01. imDCs: immature DCs; mDCs: mature DCs; tDCs: tolerogenic DCs. Supplementary Table 1: clinical characteristics of patients. Supplementary Table 2: real-time RT-PCR primer sequences.
