# Supplementary material for: IL-37 Plays a Beneficial Role in Patients with Acute Coronary Syndrome
Source: Mediators Inflamm. 2019 Oct 9;2019:9515346. doi: 10.1155/2019/9515346 (PMC6803729; doi:10.1155/2019/9515346)
Supplement: Supplementary Materials — Figure S1: the number of CD4+ cells in the four groups. All data are expressed as mean ± SEM, and one-way ANOVA was followed by a post hoc Student-Newman-Keuls test. NCA (n = 30), SA (n = 26), UA (n = 35), and AMI (n = 38). ∗ indicates P < 0.05 and ∗∗ indicates P < 0.01. NCA: normal coronary artery; SA: stable angina; UA: unstable angina; AMI: acute myocardial infarction. Figure S2: Th1-, Th17-, and Treg-related gene expression levels in the four groups. The mRNA expressions of T-bet, IFN-γ, RORγt, IL-17, Foxp3, IL-10, and TGF-β are shown. All data are expressed as mean ± SEM, and one-way ANOVA was followed by a post hoc Student-Newman-Keuls test. NCA (n = 30), SA (n = 26), UA (n = 35), and AMI (n = 38). ∗ indicates P < 0.05 and ∗∗ indicates P < 0.01. NCA: normal coronary artery; SA: stable angina; UA: unstable angina; AMI: acute myocardial infarction. Figure S3: TNI levels were positively correlated with CRP for the AMI patients (n = 38). AMI: acute myocardial infarction. Figure S4: PBMCs cultured with serum from UA or AMI patients significantly downregulated the frequencies of Tregs (CD4+Foxp3+/CD4+ T cells) and markedly increased the frequencies of Th1 (CD4+IFN-γ+/CD4+ T cells) and the proportions of Th17 (CD4+IL-17+/CD4+ T cells) compared with PBMCs cultured with serum from NCA or SA patients. All data are expressed as mean ± SEM, and one-way ANOVA was followed by a post hoc Student-Newman-Keuls test. NCA (n = 10), SA (n = 10), UA (n = 10), and AMI (n = 10). ∗∗ indicates P < 0.01. ns: not significant; NCA: normal coronary artery; SA: stable angina; UA: unstable angina; AMI: acute myocardial infarction. Figure S5: the effect of IL-37 on Th1-, Th17-, and Treg-related gene expression levels in activated PBMCs. The mRNA expressions of T-bet, IFN-γ, RORγt, IL-17, Foxp3, IL-10, and TGF-β are shown. All data are expressed as mean ± SEM, and differences were evaluated using Student's t-test. NCA (n = 30), SA (n = 26), UA (n = 35), and AMI (n = 38). ∗∗ indicates P < 0.01 [file 9515346.f1.docx]

Figure S1





Figure S1. The number of CD4+ cells in the four groups. All data are expressed as mean ± SEM and One-way ANOVA was followed by a post hoc Student-Newmann-Keuls test. NCA (n=30), SA (n=26), UA (n=35), and AMI (n=38). * indicates P<0.05 and ** indicates P<0.01. NCA: normal coronary artery; SA: stable angina; UA: unstable angina; AMI: acute myocardial infarction.

Figure S2

**

**

Figure S2. Th1, Th17 and Tregs related gene expression levels in the four groups. The mRNA expressions of T-bet, IFN-γ, RORγt, IL-17, Foxp3, IL-10 and TGF-β are shown. All data are expressed as mean ± SEM and One-way ANOVA was followed by a post hoc Student-Newmann-Keuls test. NCA (n=30), SA (n=26), UA (n=35), and AMI (n=38). * indicates P<0.05 and ** indicates P<0.01. NCA: normal coronary artery; SA: stable angina; UA: unstable angina; AMI: acute myocardial infarction.

Figure S3

**

**

Figure S3. TNI levels were positively correlated with CRP for the AMI patients (n=38). AMI: acute myocardial infarction.

Figure S4





Figure S4. PBMCs cultured with serum from UA or AMI patients significantly down-regulated the frequencies of Tregs (CD4^+^Foxp3^+^/CD4^+^ T cells), and markedly increased the frequencies of Th1 (CD4^+^IFN-γ^+^/CD4^+^ T cells) and the proportions of Th17 (CD4^+^IL-17 ^+^/CD4^+^ T cells) compared with PBMCs cultured with serum from NCA or SA patients. All data are expressed as mean ± SEM and One-way ANOVA was followed by a post hoc Student-Newmann-Keuls test. NCA (n=10), SA (n=10), UA (n=10), and AMI (n=10). ** indicates P<0.01 and ns indicates not significant. NCA: normal coronary artery; SA: stable angina; UA: unstable angina; AMI: acute myocardial infarction.

Figure S5

**

**

Figure S5. The effect of IL-37 on Th1, Th17 and Tregs related gene expression levels in activated PBMCs. The mRNA expressions of T-bet, IFN-γ, RORγt, IL-17, Foxp3, IL-10 and TGF-β are shown. All data are expressed as mean ± SEM and differences were evaluated using Student t test. NCA (n=30), SA (n=26), UA (n=35), and AMI (n=38). ** indicates P<0.01 and ns indicates not significant. NCA: normal coronary artery; SA: stable angina; UA: unstable angina; AMI: acute myocardial infarction.

Figure S6

**
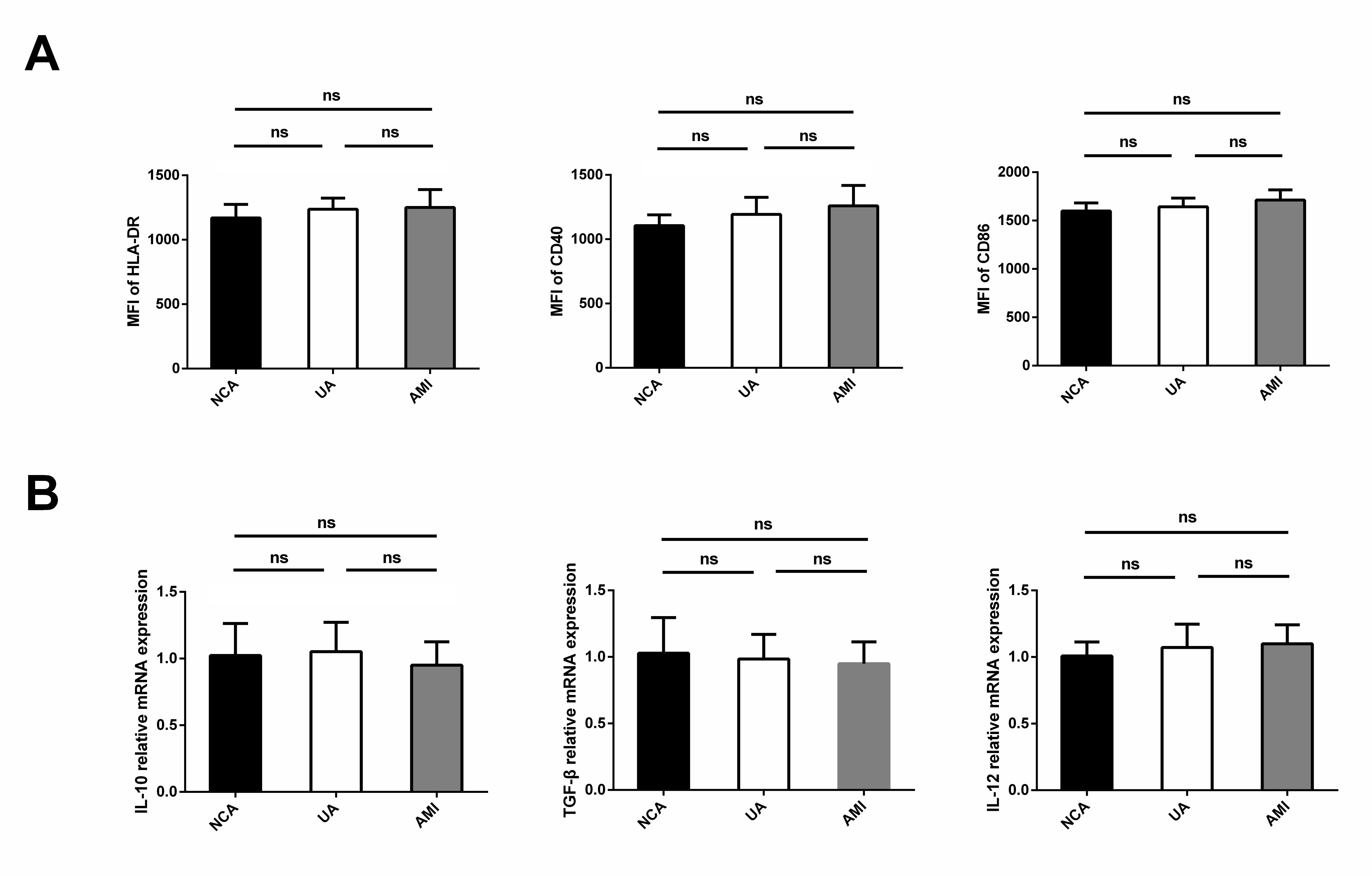
**

Figure S6. IL-37-treated DCs from patients with ACS are phenotypically and functionally comparable to IL-37-treated DCs from NCA patients. A, Mean fluorescence intensities (MFIs) for HLA-DR, CD40, and CD86 were quantified. B, Analysis of the mRNA levels of IL-10, TGF-β, and IL-12 in different patients groups. All data are expressed as mean ± SEM and One-way ANOVA was used. NCA (n=6), UA (n=8), and AMI (n=8), ns indicates not significant. NCA: normal coronary artery; UA: unstable angina; AMI: acute myocardial infarction.

Figure S7

**

**

Figure S7. TLR-4 relative mRNA expressions in the three groups were shown. All data are expressed as mean ± SEM (n = 6/experiment and three experiments were performed) and One-way ANOVA was followed by a post hoc Student-Newmann-Keuls test. ** indicates P<0.01. ImDCs= immature DCs, mDCs= mature DCs, and tDCs= tolerogenic DCs.

Figure S8

**
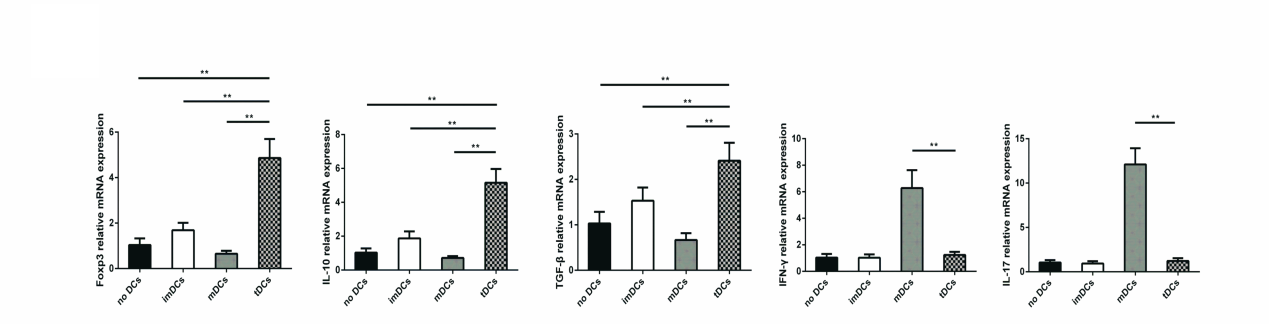
**

Figure S8. Analysis of the mRNA levels of Foxp3, IL-10, TGF-β, IFN-γ and IL-17 in different groups. All data are expressed as mean ± SEM (n = 5/experiment and three experiments were performed) and One-way ANOVA was followed by a post hoc Student-Newmann-Keuls test. ** indicates P<0.01. ImDCs= immature DCs, mDCs= mature DCs, and tDCs= tolerogenic DCs.

Supplementary Table 1: Clinical characteristics of patients

| Characteristics | NCA | SA | UA | AMI |
| --- | --- | --- | --- | --- |
|  | (n=30) | (n=26) | (n=35) | (n=38) |
| Age (years) | 57.1 ± 10.1 | 59.2 ± 10.3 | 58.3 ± 9.7 | 58.2 ± 9.9 |
| Sex(male/female) | 21/9 | 18/8 | 24/11 | 26/12 |
| Smoking, n (%) | 11 (36.7) | 10 (38.5) | 16 (45.7) | 15 (39.0) |
| Hypertension, n(%) | 19 (63.3) | 16 (61.5) | 21 (60.0) | 23 (60.5) |
| Diabetes, 𝑛 (%) | 8 (26.7) | 8 (30.8) | 13 (37.1) | 13 (34.2) |
| TC (mmol/L) | 3.96 ± 0.99 | 4.32 ± 0.78 | 4.13 ± 0.94 | 4.30 ± 1.38 |
| TG (mmol/L) | 1.56 ± 0.99 | 1.90 ± 1.10 | 1.63 ± 0.78 | 1.52 ± 0.72 |
| LDL-C (mmol/L) | 2.19 ± 0.73 | 2.54 ± 0.88 | 2.35 ± 0.69 | 2.38 ± 1.03 |
| HDL-C (mmol/L) | 1.21 ± 0.38 | 1.24 ± 0.30 | 1.19 ± 0.30 | 1.08 ± 0.35 |
| GLU (mmol/L) | 5.13 ± 0.88 | 5.18 ± 1.03 | 5.56 ± 1.43 | 5.83 ± 1.88 |
| Creatinine (𝜇mol/L) | 72.66 ± 21.83 | 73.33 ± 16.60 | 72.95 ± 23.19 | 98.53 ± 45.17**^##&&^ |
| hs-CRP (mg/L) | 3.37 ± 2.51 | 3.81 ± 2.40 | 7.43 ± 4.91* | 11.28 ± 8.62**^##&^ |
| cTnI (pg/mL) | 7.10 ± 11.03 | 7.70 ± 8.68 | 8.89 ± 11.94 | 5772.1 ± 7364.5**^##&&^ |
| Medications, 𝑛 (%) |  |  |  |  |
| Aspirin | 6 (20.0) | 13 (50.0) | 26 (74.3) | 22 (57.9) |
| β- blocker | 8 (26.7) | 9 (34.6) | 14 (40.0) | 13 (34.2) |
| ACEI/ARB | 14 (46.7) | 10 (38.5) | 16 (45.7) | 20 (52.6) |
| CCB | 17 (56.7) | 13 (50.0) | 21 (60.0) | 12 (31.6) |
| Statin | 9 (30) | 17 (65.4) | 24 (68.6) | 18 (47.4) |

The data are given as the mean ± SD or number of patients. NCA: normal coronary artery; SA: stable angina; UA: unstable angina; AMI: acute myocardial infarction; TC: total cholesterol; TG: total triglycerides; LDL-C: low-density lipoprotein cholesterol; HDL-C: high-density lipoprotein cholesterol; GLU: fasting glucose; hs-CRP: high sensitive C-reactive protein; cTnI: Cardiac Troponin I; ACEI: angiotensin-converting enzyme inhibitor; ARB: angiotensin receptor blocker; CCB: calcium channel blocker.

*P<0.05 versus NCA, **P< 0.01 versus NCA, ^#^P<0.05 versus SA, ^##^P< 0.01 versus SA, ^&^P<0.05 versus UA, and ^&&^P< 0.01 versus UA.

Supplementary Table 2 Real-time RT-PCR Primer Sequences

| ***Gene*** | ***Forward (5’-3’)*** | ***Reverse (5’-3’)*** |  |
| --- | --- | --- | --- |
| T-bet | TTGAGGTGAACGACGGAGAG | CCAAGGAATTGACAGTTGGGT | |
| IFN-γ | TCGGTAACTGACTTGAATGTCCA | TCGCTTCCCTGTTTTAGCTGC | |
| RORγt | GTGGGGACAAGTCGTCTGG | AGTGCTGGCATCGGTTTCG | |
| IL-17A | TCCCACGAAATCCAGGATGC | GGATGTTCAGGTTGACCATCAC | |
| IL-12p40 | TGCCCATTGAGGTCATGGTG | CTTGGGTGGGTCAGGTTTGA | |
| Foxp3 | GTGGCCCGGATGTGAGAAG | GGAGCCCTTGTCGGATGATG | |
| IL-10 | TCAAGGCGCATGTGAACTCC | GATGTCAAACTCACTCATGGCT | |
| TGF-β  TLR-4 | GGCCAGATCCTGTCCAAGC  AGCCGCTGGTGTATCTTTGA | GTGGGTTTCCACCATTAGCAC  GCATCCTGTACCCACTGTTC | |
| GAPDH | CTGGGCTACACTGAGCACC | AAGTGGTCGTTGAGGGCAATG | |

All these primers were synthesized by Tsingke in Wuhan, China.
